# Promoting Surface Energy and Osteoblast Viability on Zirconia Implant Abutments Through Glass–Ceramic Spray Deposition Technology

**DOI:** 10.3390/jfb16080288

**Published:** 2025-08-07

**Authors:** Wen-Chieh Hsu, Tao-Yu Cha, Yu-Chin Yao, Chien-Ming Kang, Sheng-Han Wu, Yuichi Mine, Chien-Fu Tseng, I-Ta Lee, Dan-Jae Lin, Tzu-Yu Peng

**Affiliations:** 1 School of Dentistry, College of Oral Medicine, Chung Shan Medical University, Taichung 40201, Taiwan; 2 Center for Tooth Bank and Dental Stem Cell Technology, College of Oral Medicine, Taipei Medical University, Taipei 11031, Taiwan; 3 Department of Oral Hygiene, Hsin Sheng Junior College of Medical Care and Management, Taoyuan 32544, Taiwan; 4 Department of Dentistry, Taoyuan General Hospital, Ministry of Health and Welfare, Taoyuan 33004, Taiwan; 5 School of Dentistry, College of Oral Medicine, Taipei Medical University, Taipei 11031, Taiwan; 6 Huayi Dental Laboratory, Taipei 10491, Taiwan; 7 Department of Medical Systems Engineering, Graduate School of Biomedical and Health Sciences, Hiroshima University, Hiroshima 734-8553, Japan; 8 Graduate Institute of Clinical Dentistry, School of Dentistry, College of Medicine, National Taiwan University, Taipei 100233, Taiwan; 9 Department of Biomedical Engineering, College of Biomedical Engineering, China Medical University, Taichung 40402, Taiwan; 10 School of Dentistry, College of Dentistry, China Medical University, Taichung 40402, Taiwan

**Keywords:** zirconia, dental restoration, abutments, glass-ceramic, spray deposition, cell behavior, live/dead staining

## Abstract

Zirconia is used widely for high-precision custom abutments; however, stress concentration can compromise osseointegration. Although glass–ceramic spray deposition (GCSD) can enhance the surface properties of zirconia, its biological effects remain unclear. In this study, the biological responses of human osteoblast-like (MG-63) cells to GCSD-modified zirconia surfaces were evaluated to assess the potential application in zirconia abutments. Disk-shaped zirconia and titanium alloy samples were prepared; titanium served as the control (Ti). Zirconia was subjected to polishing (NT), airborne-particle abrasion (AB), or GCSD with (GE) or without (GC) hydrofluoric acid (HF) etching. Surface characteristics, including wettability, surface energy (SE), and surface potential (SP), were analyzed. Cytotoxicity and MG-63 cell adhesion were assessed using the PrestoBlue assay, scanning electron microscopy (SEM), viability staining, and confocal laser scanning microscopy (CLSM). Statistical analysis was performed with a significance level of 0.05. GCSD produced a dense glass–ceramic coating on the zirconia surface, which significantly enhanced hydrophilicity as indicated by reduced water contact angles and increased SE in the GC and GE groups (*p* < 0.05). HF etching increased SP (*p* < 0.05). No cytotoxicity was observed in any group. SEM, viability staining, and CLSM revealed enhanced MG-63 cell attachment on Ti and GE surfaces and the highest viability ratio in the GE group. The NT group exhibited the lowest cell attachment and viability at all time points. GCSD effectively improved zirconia abutment surface properties by enhancing hydrophilicity and promoting MG-63 cell adhesion, with biocompatibility comparable to or better than that of titanium.

## 1. Introduction

Zirconia has become a material of choice in modern dental restorations due to its outstanding mechanical strength, fracture toughness, and chemical stability, which enable it to endure the complex and repetitive stresses encountered in the oral environment [1,2]. Its application is particularly prevalent in the fabrication of high-precision custom abutments, where both biomechanical performance and esthetic outcomes are critically important. In comparison to conventional titanium or other metal-based abutments, zirconia abutments exhibit superior optical properties, such as natural translucency and color matching, thereby providing improved esthetic integration in anterior restorations. Moreover, their smoother surface and lower surface energy contribute to reduced bacterial adhesion, subsequently decreasing the incidence of peri-implant inflammation, including gingivitis and peri-implant mucositis [1,3]. Despite these advantages, the biological inertness and relatively poor interfacial affinity of zirconia to both hard and soft tissues remain significant limitations. The lack of functional groups on the zirconia surface results in suboptimal protein adsorption and inadequate cell attachment, which are essential processes for early tissue integration. Furthermore, its intrinsically smooth and dense microstructure, while beneficial for mechanical integrity, compromises bonding efficacy with resin-based adhesives and hampers epithelial or connective tissue sealing. These challenges have driven increasing research interest in developing advanced surface treatment strategies, such as selective etching, coating, or energy-based modifications, that can alter surface topography, increase surface energy, or introduce bioactive chemical functionalities to promote stronger adhesion and enhanced biological responses. Additionally, the high elastic modulus of zirconia, which far exceeds that of bone, may contribute to the concentration of occlusal and masticatory forces at the bone–implant interface. This mismatch in mechanical properties can result in stress transmission directly to the alveolar bone or implant fixture, potentially leading to biomechanical complications [4,5,6]. Over time, the stress-shielding effect induced by zirconia’s rigidity may reduce the mechanical stimulation required for bone remodeling, thereby compromising bone homeostasis. Clinical consequences include marginal bone loss, a decrease in local bone mineral density, and an elevated risk of implant-related bone resorption or structural failure of the abutment [7,8]. Therefore, improving the interfacial properties of zirconia through surface modification is crucial to enhancing its clinical performance and long-term stability.

Treatment strategies for dental implant abutment surfaces are often tailored based on their anatomical location and functional requirements. In the transmucosal region, where the abutment traverses the peri-implant soft tissue, surface treatments are primarily aimed at minimizing bacterial adhesion and plaque accumulation to preserve soft tissue health [9,10]. In this context, mechanical polishing and other finishing techniques are frequently employed to produce a smooth, bioinert surface that discourages microbial colonization and facilitates effective oral hygiene maintenance [11]. Conversely, in the cementation region, where the abutment interfaces with the prosthetic crown or restoration, surface roughening is typically required to enhance micromechanical interlocking and improve the durability of the adhesive bond. Techniques such as airborne-particle abrasion, sandblasting with alumina particles, and laser irradiation are commonly utilized to increase surface roughness and surface energy, thereby promoting better retention of the prosthetic components and ensuring long-term clinical success [12,13]. At the implant–abutment connection, which plays a vital role in achieving and maintaining osseointegration, additional surface modifications are often applied to stimulate osteogenic responses. Treatments such as acid etching, anodization, or the incorporation of bioactive coatings have been reported to improve osteoblast attachment, proliferation, and bone-to-implant contact, contributing to greater mechanical stability and long-term implant survival [14,15,16]. Although these region-specific strategies are effective in optimizing the biological and mechanical performance of the implant system, they inevitably introduce procedural complexity and prolong chairside time. This challenge is further exacerbated in fully digital workflows, particularly when using prefabricated zirconia abutments, where access to individual surface regions is limited and selective treatment becomes difficult to implement. As such, the development of a standardized surface treatment protocol that can simultaneously meet the diverse functional demands across all relevant regions, such as promoting reliable bonding to prosthetic materials, enhancing surface wettability, and supporting favorable tissue responses, would offer significant clinical advantages. Such a universal approach could simplify restorative procedures, reduce treatment time, and enhance the predictability and reproducibility of implant-based rehabilitations.

Glass–ceramic spray deposition (GCSD) represents a novel and promising surface treatment technique for zirconia, offering significant improvements in both mechanical and biological performance. This method modifies the zirconia surface by simultaneously increasing surface roughness and enhancing wettability, which in turn improves bonding strength and promotes better mechanical integration with surrounding tissues and restorative materials [17,18]. In this approach, a lithium disilicate-based glass–ceramic aerosol is prepared and applied onto zirconia substrates. The glass–ceramic precursor is formulated by combining silica sand, lithium oxide, and various other metal oxides, such as sodium oxide, alumina, and zirconia, in carefully controlled proportions. The blended materials are melted at high temperatures, rapidly quenched to form amorphous frits, and then mechanically milled into fine powders. These powders are subsequently suspended in a volatile medium to create a sprayable aerosol. Upon application, the aerosol is uniformly sprayed onto the zirconia surface and subjected to a heat treatment process, which facilitates the formation of a dense and adherent lithium disilicate glass–ceramic coating [17]. This surface layer serves multiple functional purposes. Mechanically, it enhances micromechanical interlocking due to the induced micro-roughness, thereby improving adhesive retention. Chemically, the coating introduces a reactive and polar interface characterized by increased surface energy, which is advantageous for both adhesive bonding and cellular interactions. The incorporation of lithium disilicate is particularly noteworthy, as this material is well-established in restorative dentistry for its excellent esthetic properties, bioactivity, and favorable interactions with soft and hard tissues. By integrating lithium disilicate into the zirconia surface, the treatment effectively combines the mechanical durability of zirconia with the biological affinity and esthetic potential of glass–ceramics [19]. Although the enhancements in surface topography, wettability, and bonding performance associated with GCSD-treated zirconia have been increasingly validated in the literature, data concerning the biological responses, such as cytocompatibility, protein adsorption, and long-term tissue integration, remain relatively limited [17,18,20,21]. Further investigations are warranted to fully elucidate the biofunctional implications of this surface modification and to support its translation into routine clinical practice.

To advance the clinical applicability of GCSD, it is important to assess not only its physical and chemical benefits but also its effects on cellular behavior. Early interactions between osteoblasts and implant surfaces play a critical role in osseointegration, and surface properties such as wettability, energy, and nanoscale topography directly influence cell adhesion, viability, and differentiation. Human osteoblast-like MG-63 cells are widely used as an in vitro model to evaluate the biological response to implant materials. Despite their tumor origin, they retain key osteoblastic features, including alkaline phosphatase activity and collagen secretion, and respond sensitively to changes in surface chemistry and roughness. Their reproducibility and stability make them suitable for preliminary biocompatibility testing. This study aimed to investigate how GCSD modifies zirconia surface characteristics and influences MG-63 cell responses. The null hypothesis was that GCSD would have no effect on cell behavior. By correlating surface parameters, including contact angle, surface potential, and elemental composition, with cellular outcomes, this work provides insight into how GCSD modulates the zirconia–cell interface. The results may support the development of zirconia abutments with improved bioactivity and long-term clinical performances.

## 2. Materials and Methods

### 2.1. Sample Preparation and Surface Treatments

**Figure 1:**
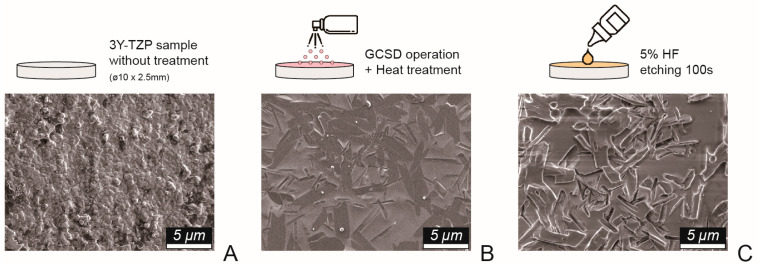
Surface treatment processes. (**A**) Zirconia (3Y-TZP) samples with a diameter of 10 mm and thickness of 2.5 mm were fabricated using a milling machine. (**B**) The samples were coated with glass–ceramic via glass–ceramic spray deposition (GCSD), followed by heat treatment (drying at 450 °C for 1 min and tempering at 895 °C for 1.5 min). (**C**) Finally, the samples were etched with 5% hydrofluoric acid (HF) for 100 s. All images were taken at ×5000 magnification; scale bar = 5 μm.

Disk-shaped zirconia specimens (Superfect Zir, Aidite Technology, Qinhuangdao, China) and grade 5 titanium alloy samples (ASTM F136, Green Dentech, Tainan, Taiwan), each with a diameter of 10 mm and thickness of 3 mm, were fabricated using a dental computer-aided design/manufacturing system. All samples were polished in a stepwise manner using #320-, #400-, and #600-grit abrasive papers, followed by ultrasonic cleaning in deionized water and air-drying. The titanium samples (Ti) served as the control group, while the zirconia specimens were randomly allocated into groups based on surface treatment (Figure 1): no further treatment (NT), airborne-particle abrasion using 50-μm aluminum oxide particles (Cobra, Renfert, Hilzingen, Germany) (AB), and glass–ceramic coating via GCSD (Biomic Lisi Connect, Aidite Technology, Qinhuangdao, China). The GCSD group was further divided into two subgroups: with etching using 5.0% hydrofluoric acid gel (IPS Ceramic Etching Gel, Ivoclar Vivadent, Schaan, Liechtenstein) for 100 s (GE), and without etching (GC).

### 2.2. Surface Characterization

Surface wettability was measured using a contact angle goniometer (Phoenix Mini, Surface Electro Optics, Suwon-si, Republic of Korea) with distilled water as the polar solvent and diiodomethane as the non-polar solvent. Each contact angle value represents the mean of 10 independent measurements. To ensure consistency, all measurements were conducted at room temperature (25 ± 1 °C) and under controlled humidity (50–60%). Before testing, specimens were ultrasonically cleaned in ethanol and air-dried to remove potential surface contaminants that could interfere with droplet spreading dynamics. Surface energy (SE) was calculated using the Owens–Wendt–Rabel–Kaelble (OWRK) method and Surfaceware software (v9, Surface Electro Optics Co.). The OWRK method provides a widely accepted approach to decompose total surface energy into its polar and dispersive components based on the contact angles of polar and non-polar liquids. This method allows for quantifying the material’s affinity toward hydrophilic versus hydrophobic interactions, which is directly relevant to protein adsorption and initial cell attachment in biological contexts. Increased SE, particularly in the polar component, is known to enhance integrin-mediated adhesion and promote favorable cell–material interactions at the tissue interface. Surface potential (SP) was measured using Kelvin probe force microscopy (KPFM; Dimension Icon VT-1000, Bruker, Bremen, Germany), calibrated with a gold standard (work function 5.1 V). Scans were performed in tapping mode over a 1 μm^2^ area to reduce topographic artifacts. SP reflects the local work function difference between the probe and zirconia surface, serving as an indicator of surface charge distribution, which can influence protein adsorption and cell behavior.

### 2.3. Cell Culture and Cytotoxicity Testing

MG-63 human osteoblast-like cells (#60279, Biosource Collection and Research Center) were cultured in Minimum Essential Medium (MEM; Invitrogen, Carlsbad, CA, USA), supplemented with 10% fetal bovine serum (FBS; Gibco, Waltham, MA, USA), 1% L-glutamine, and 1% penicillin-streptomycin solution. Cultures were incubated at 37 °C in a humidified incubator containing 5% CO_2_, and the medium was replaced every 2–3 days to maintain optimal nutrient conditions. For cytotoxicity testing, autoclaved test material specimens (121 °C, 1.2 bar, 30 min) were immersed in MEM supplemented with antibiotics and incubated at 37 °C for 72 h to prepare extracts. MG-63 cells were seeded in 96-well plates at a density of 1 × 10^4^ cells/well and allowed to attach overnight. Subsequently, the culture medium was replaced with 100 μL of the prepared extracts, and cells cultured in standard MEM served as the negative control. Cell viability was assessed at 1, 2, and 3 days using PrestoBlue Cell Viability Reagent (Thermo Fisher Scientific, Waltham, MA, USA), according to the manufacturer’s instructions. All assays were performed in triplicate, and viability was expressed as a percentage relative to the control group.

### 2.4. Cell Adhesion

For cell adhesion analysis, sterilized test specimens were placed in 6-cm culture dishes and seeded with MG-63 cells for 4 h. After incubation, the samples were rinsed with phosphate-buffered saline, fixed with 4% paraformaldehyde for 15 min at room temperature, followed by de-hydration in a graded ethanol series (30%, 50%, 70%, 90%, and 100%, 10 min each step). The dehydrated samples were then subjected to critical point drying (CPD030, Bal-Tec, Balzers, Liechtenstein), then sputter-coated with a 10-nm-thick platinum layer using an automatic fine coater (JEOL JEC-3000FC, Tokyo, Japan) at 10 mA for 25 s. The cell morphology, spreading, and density on the sample surfaces were examined using field-emission scanning electron microscopy (FE-SEM; JSM-7800F Prime, JEOL) operated at 3.0 kV under high-vacuum conditions (9.6 × 10^−5^ Pa). Images were acquired from at least three randomly selected fields per sample to ensure representative evaluation.

### 2.5. Live/Dead Staining and Confocal Laser Scanning Microscopy (CLSM)

To further assess cell viability and membrane integrity, live/dead staining was performed using the Calcein AM/Ethidium Homodimer-1 assay kit (LIVE/DEAD™ Viability/Cytotoxicity Kit, Invitrogen). MG-63 cells were seeded onto the sterilized samples placed in 24-well plates at a density of 5 × 10^4^ cells/well and cultured for 1 and 3 days. After incubation, cells were gently washed with PBS and incubated in a staining solution containing 2 μM Calcein AM and 4 μM EthD-1 in PBS for 30 min at 37 °C, protected from light. Fluorescence images were captured using a confocal laser scanning microscope (CLSM; Leica TCS SP2, Wetzlar, Germany). Calcein AM-stained live cells (green) and EthD-1-stained dead cells (red) were visualized at 488 nm and 568 nm excitation wavelengths, respectively. Quantitative analysis of live and dead cell ratios was conducted using ImageJ software (ver 1.54; NIH, Bethesda, MD, USA) with the Color Pixel Counter plugin to calculate relative pixel areas of fluorescence. Three independent images were analyzed per sample.

### 2.6. Statistical Analyses

Sample sizes were calculated using power analysis (G*Power v3.1.9.6) to ensure a power greater than 0.9 at an alpha level of 0.05. Statistical analyses were conducted using SPSS (v19; IBM, Armonk, NY, USA) and GraphPad Prism (v10; GraphPad Software, La Jolla, CA, USA). The Shapiro–Wilk test was used to verify normality. One-way analysis of variance (ANOVA) followed by Tukey’s honestly significant difference (HSD) post hoc test was used to evaluate differences between groups. The level of statistical significance was set at *p* < 0.05.

## 3. Results

### 3.1. Surface Wettability and Potential Evaluation

**Figure 2:**
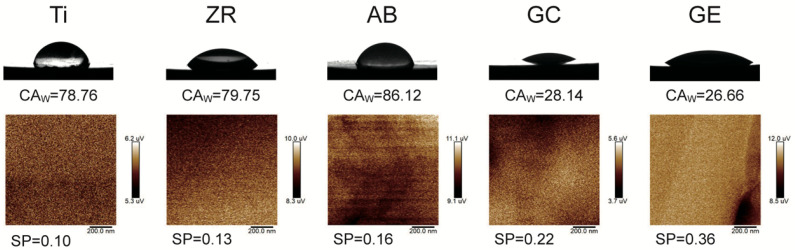
Results of surface wettability analysis measured by the contact angle (degree) and surface potential measured by Kelvin probe force microscopy (µV).

The microstructural analysis shown in Figure 1 reveals that GCSD produced platelet-like lithium silicate and rod-like lithium disilicate crystal structures on the zirconia surface. HF etching dissolved the glassy phase, exposing distinct, interwoven lithium disilicate structures. The results of surface characterization are presented in Figure 2 and Table 1. In the absence of GCSD treatment (Ti, ZR, and AB groups), the Ti group exhibited relatively lower contact angles with both water (CA_W_) and diiodomethane (CA_D_), as well as lower SP and higher SE, although these differences were not statistically significant within this subgroup. In contrast, the GC and GE groups, which underwent GCSD treatment, demonstrated a significant reduction in CA_w_ and a marked increase in SE (*p* < 0.05). This suggests that the formation of a glass–ceramic interfacial layer effectively enhanced surface hydrophilicity and surface energy. Furthermore, the GE group, which received both GCSD and HF etching, showed the highest SP value (0.36 ± 0.14 μV), significantly greater than all other groups, indicating a strong alteration in surface charge properties likely due to the exposure of crystalline disilicate structures.

### 3.2. Cell Cytotoxic and SEM Evaluation of Cell Morphology

**Figure 3:**
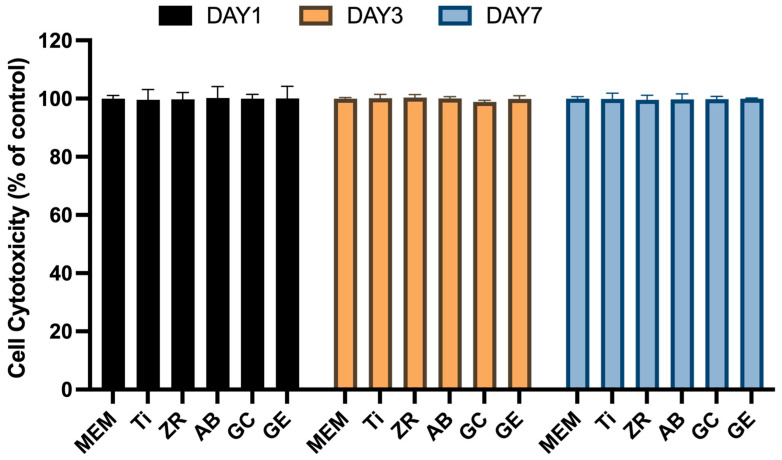
Assessment of cell cytotoxicity. The cytotoxicity of the tested samples was evaluated by indirectly exposing MG-63 cells to sample extracts, followed by the PrestoBlue assay.

**Figure 4:**
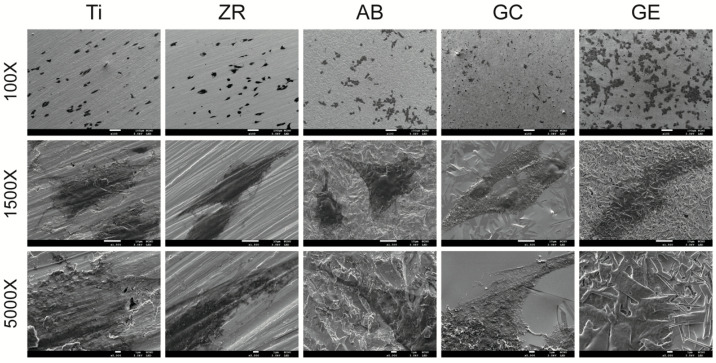
FE-SEM micrographs of MG-63 cell adhesion. FE-SEM images obtained after 4 h show adherent MG-63 cells on the surfaces of the testing samples (magnification: ×100, ×1500, and ×5000).

To evaluate the cytotoxic potential of the surface treatments, cell viability assays were conducted (Figure 3). None of the surface-treated groups exhibited statistically significant cytotoxicity compared with the MEM control, regardless of the culture duration (*p* > 0.05). Additionally, no significant differences in cell viability were observed among the treatment groups (*p* > 0.05), indicating that none of the surface modifications exerted cytotoxic effects. Figure 4 presents SEM micrographs of MG-63 cells cultured for 4 h, which reflect the initial cell adhesion behavior. Low-magnification (×100) images show that the cell density was highest on the GE surface and lowest on the GC surface. At intermediate (×1500) and high magnifications (×5000), pseudopodia extensions were visible on the ZR, AB, and GC surfaces, while denser cell attachment was evident on the Ti and GE surfaces.

### 3.3. Live/Dead Fluorescent Staining and CLSM

**Figure 5:**
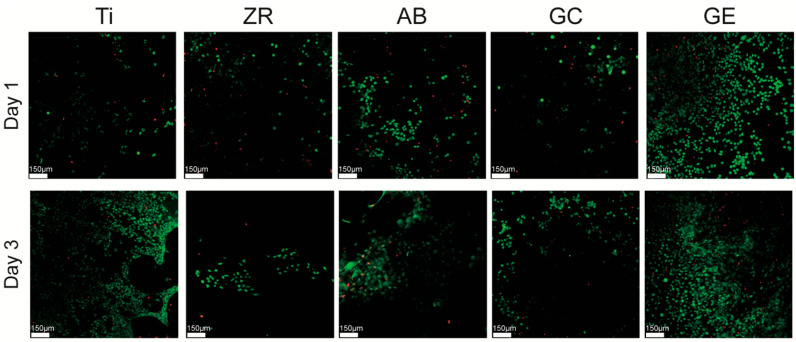
Viability assay of MG-63 cells cultured on testing samples for 1 and 3 days. Live cells are indicated by green fluorescence (Calcein-AM), while dead cells exhibit red fluorescence (ethidium homodimer-1). Scale bars represent 150 µm.

Viability staining analysis via CLSM was used to further assess cell viability under various culture conditions (Figure 5). The ratio of live cells was calculated based on the ratio of green fluorescence to total fluorescence. After 1 day of culture, the GE and ZR groups showed the highest and lowest live cell areas, respectively. After 3 days, all groups demonstrated an increase in the live cell area. The area in Ti culture reached a level comparable to that in GE culture; the levels in ZR, AB, and GC culture remained significantly lower, with ZR consistently displaying the lowest viability.

## 4. Discussion

The findings of this study confirm that GCSD can significantly enhance the surface hydrophilicity and SP of zirconia, while reduce SE and promote the adhesion of human osteoblast-like cells. The comparable or even superior cell viability observed on GCSD-treated zirconia relative to titanium supports the rejection of the null hypothesis.

GCSD is a straightforward and intuitive surface treatment technique that requires only subsequent heat treatment and acid etching to form a dense and uniform lithium disilicate glass–ceramic coating on the zirconia surface [17,19]. Peng et al. [17] showed that 100 s HF etching effectively exposed lithium disilicate and enhanced surface properties without damaging the coating; thus, this condition was adopted in the present study. Jin [20] and Shen [21] et al. demonstrated that GCSD improves the surface wettability and roughness of zirconia, and these findings are corroborated by the current experimental results (Table 1). Furthermore, Liang et al. [19] reported that the thin glass–ceramic coating produced by GCSD makes it a clinically viable method for zirconia surface modification. Consequently, the application of GCSD to implant abutments is unlikely to cause compression or adverse effects on the surrounding peri-implant gingival tissue. Cytotoxicity can negatively impact implant performance and may lead to unfavorable tissue responses, failure of osseointegration, or even implant loss [15,16]. The current results confirm that GCSD did not induce cellular cytotoxicity (Figure 3), indicating that it is safe for clinical applications. However, Peng et al. [17] also noted that over-etching may damage the surface texture. Therefore, HF etching after GCSD should be conducted with caution, and thorough rinsing after etching is essential.

Surface energy plays a crucial role in determining the biocompatibility of materials. This study demonstrated that GCSD could generate micron-scale surface structures that effectively alter both SE and hydrophilicity [17]. Untreated zirconia exhibited an SE of 37 mN/m, while GCSD doubled this value to over 78 mN/m. In terms of hydrophilicity, the contact angle decreased significantly from 80° to below 30° (*p* < 0.05). Razafiarison et al. [22] indicated that SE modulates cell adhesion and differentiation on material surfaces, with hydrophobic surfaces suppressing cellular mechanosensitivity. Similarly, Kirchhof et al. [23] reported that materials with higher SE promote bioadhesion to a greater extent than those with lower SE. In the current study, SEM revealed that the GE group had a greater number of adherent cells (Figure 4) and a higher proportion of live cells (Figure 5). In contrast, the GC group produced inferior biological responses, which are likely to be due to lower SP. Metwally et al. [24] emphasized that SP is a key regulator of cellular responses, as it mediates adhesion and signaling pathways for tissue regeneration. Chang et al. [25] reported that an increase in SP is associated with enhanced cell adhesion. In the current study, the GE group demonstrated significantly higher SP values than the other groups (Table 1 and Figure 3), which may account for the superior MG-63 cell responses elicited by this sample. In addition, the surface energy values reported in Table 1 were calculated using the OWRK method, which distinguishes between polar and dispersive (non-polar) components based on the contact angles of both polar (distilled water) and non-polar (diiodomethane) liquids. The GE group exhibite the highest polar component among all groups, suggesting an increase in surface polarity and hydroxyl group exposure after HF etching. These changes are known to favor protein adsorption and cell adhesion. By analyzing the separate contributions of polar and dispersive components, we were able to better understand the nature of the surface activity introduced by GCSD. This more detailed assessment strengthens the interpretation that the enhanced biological responses observed in the GE group are associated with both chemical and topographical surface modifications [22,23,24,25].

Surface potential (SP) plays a critical yet often underappreciated role in mediating early biological responses on biomaterial surfaces [17]. Most mammalian cells, including osteoblasts, possess negatively charged membranes due to the presence of phospholipids and sialic acid residues. This creates a favorable electrostatic interaction with surfaces exhibiting moderate positive SP, which can enhance initial protein adsorption, promote integrin-mediated adhesion, and stimulate downstream signaling pathways related to proliferation and osteogenic differentiation [23,24,25]. In addition, surface potential gradients have been shown to modulate local ionic environments, affecting calcium and phosphate attraction, which is beneficial for mineralization and osseointegration. In the present study, the GE group demonstrated significantly higher SP values compared to other groups, which, along with its enhanced polar surface energy and reduced contact angle, may explain its superior performance in supporting cell viability and adhesion. These findings suggest that tuning SP through surface modification not only enhances surface wettability but also contributes to the electrochemical compatibility of zirconia with biological systems [26].

Titanium was chosen as the control in this study because it is used extensively in dental implantology. Although metal-free restoration is increasingly used in clinical settings, titanium remains widely used and is considered the gold-standard material for dental implants. This is largely attributed to its excellent biocompatibility and osseointegration capacity [5,14]. Upon implantation, titanium rapidly forms a dense and stable titanium dioxide layer on its surface that is highly resistant to dissolution under physiological conditions. This oxide layer also reduces cytotoxicity and promotes interactions with osteoblasts and extracellular matrix proteins, thereby enhancing cell adhesion and differentiation [27,28]. Additionally, the titanium surface can bind calcium and phosphate ions, promoting the formation of hydroxyapatite-like structures that further support osseointegration [29]. In the current study, however, GCSD combined with HF etching significantly improved the biological properties of zirconia. The treated zirconia demonstrated cellular responses comparable to or better than those of titanium in terms of cytotoxicity (Figure 3), cell adhesion (Figure 4), and cell viability (Figure 5). Note that, surface roughness (Ra) and SE play critical roles in cell adhesion and proliferation. Recent studies have shown that nanoscale roughness and moderate surface energy (~70 mN/m) promote protein adsorption and enhance cellular attachment and proliferation [30]. In our previous study [17] atomic force microscopy revealed a significant increase in Ra after HF etching, reaching 0.151 μm. This increase in Ra was accompanied by enhanced surface area and topographic complexity, which facilitates micromechanical interlocking and provides more binding sites for proteins and cells. Although topographical analysis was not repeated in the current study, the observed SP distribution and decreased contact angle values indicate a synergistic relationship between Ra, SE, and wettability. The enhanced SE measured by KPFM is likely attributed to both the increased nanoscale roughness and the formation of a polar, chemically active surface. Therefore, the favorable cellular responses observed in this study can be attributed to treatment-induced modifications in both Ra and SE. Furthermore, SP influences electrostatic interactions between biomaterials and negatively charged cell membranes, promoting protein adsorption, cell adhesion, and signaling. The elevated SP in the GE group likely contributed to the favorable osteoblast responses observed in this study [31].

Based on the findings of this study, GCSD-treated zirconia abutments present a promising surface modification strategy to support peri-implant tissue integration. The lithium disilicate coating significantly improves surface energy and hydrophilicity, facilitating protein adsorption and early cell adhesion [20,32]. In addition, its relatively lower elastic modulus and microstructural characteristics may help buffer functional stress at the implant–abutment interface, potentially reducing marginal bone loading. Clinically, this multifunctional surface supports both hard and soft tissue responses, contributing to enhanced implant stability and long-term success [33,34]. Its clinical application may be limited by the technique sensitivity of the GCSD process and the need for cautious handling of HF during surface etching. Nevertheless, it remains unclear whether the glass–ceramic-coated zirconia surface can effectively bind calcium and phosphate ions to form a hydroxyapatite-like layer [35]. Systematic investigations are required to elucidate its potential contribution to osseointegration. Moreover, the absence of in vivo experiments limits the ability to assess long-term implant stability and clinical outcomes. This study also did not evaluate the mechanical contributions or potential shock-absorbing effects of the glass–ceramic coating under load-bearing conditions, nor did it examine long-term cell proliferation, focal adhesion, osteogenic differentiation, or biological stability over time. These aspects warrant further investigation through mechanical testing and in vivo studies to fully clarify the clinical relevance and biofunctionality of GCSD-treated zirconia surfaces.

## 5. Conclusions

The GCSD does not increase the cytotoxicity of zirconia but significantly enhances its surface hydrophilicity and surface potential. The GCSD-treated zirconia surfaces also promote human osteoblast-like cell adhesion and maintain cell viability at levels comparable to or greater than those observed on titanium surfaces. These findings suggest that GCSD is a promising surface treatment strategy for zirconia abutments, with potential to improve biological integration in clinical applications

## Figures and Tables

**Table 1:** Analysis of surface characteristics.

Group	CA_W_ (Degree)	CA_D_ (Degree)	SE (mN/m)	SP (µV)
Ti	78.76 ± 1.96 ^a^	34.91 ± 1.68 ^a^	37.86 ± 1.48 ^a^	0.10 ± 0.01 ^a,b^
ZR	79.75 ± 1.03 ^a^	35.66 ± 2.80 ^a^	37.09 ± 1.13 ^a^	0.13 ± 0.01 ^a,b^
AB	86.12 ± 1.36 ^b^	37.51 ± 2.14 ^a^	33.15 ± 1.11 ^a^	0.16 ± 0.01 ^a^
GC	28.14 ± 2.26 ^c^	47.11 ± 5.55 ^b^	78.02 ± 1.67 ^b^	0.22 ± 0.04 ^b^
GE	26.66 ± 1.16 ^c^	44.61 ± 4.57 ^b^	79.01 ± 0.76 ^b^	0.36 ± 0.14 ^c^

CA_W_, contact angle of distilled water (polar solvent); CA_D_, contact angle of diiodomethane (non-polar solvent); SE, surface energy calculated using the Owens–Wendt method; SP, surface potential measured using Kelvin probe force microscopy. Different superscript letters (a, b, c) indicate a significant between-group difference in the mean of the parameter (*p* < 0.05).

## Data Availability

The original contributions presented in the study are included in the article further inquiries can be directed to the corresponding author.
